# Supplementary material for: Combined Cytokine Blockade Therapy (CCBT) Using Basiliximab and Infliximab for Treatment of Steroid-Refractory Graft-Versus-Host Disease (SR-GvHD)
Source: Cancers (Basel). 2024 Nov 22;16(23):3912. doi: 10.3390/cancers16233912 (PMC11640275; doi:10.3390/cancers16233912)
Supplement: Supplementary file 1 [file cancers-16-03912-s001.zip › cancers-3269605-supplementary.pdf]

**Supplemental Table S1: Univariate Analysis**							
			Response at Day 28				
		N	*CR or PR*	*Non-responder*	*Exact p value*	*Exact OR (95%CI)**	*Exact test p value**
Age at HCT, years	≤59	38	17(44.7%)	21(55.3%)	0.27	Reference	0.37
	≥60	22	6(27.3%)	16(72.7%)		0.49(0.12,1.85)	
Recipient sex	M	39	18(46.2%)	21(53.8%)	0.10	Reference	0.39
	F	21	5(23.8%)	16(76.2%)		0.47(0.10,1.97)	
Donor age, years	≤29	31	12(38.7%)	19(61.3%)	1.00	Reference	1.00
	≥30	29	11(37.9%)	18(62.1%)		1.17(0.34,4.13)	
HCT-CI	0	11	5(45.5%)	6(54.5%)	0.22	Reference	0.30
	1-2	21	5(23.8%)	16(76.2%)		0.29(0.03,2.16)	
	≥3	28	13(46.4%)	15(53.6%)		1.09(0.19,6.58)	
KPS	90-100	41	19(46.3%)	22(53.7%)	0.088	Reference	0.12
	≤80	19	4(21.1%)	15(78.9%)		0.30(0.06,1.26)	
DRI	Low, Intermediate, non-Malignant	42	17(40.5%)	25(59.5%)	0.77	Reference	0.61
	High, Very High	18	6(33.3%)	12(66.7%)		0.59(0.12,2.41)	
Female to Male HCT	No	49	16(32.7%)	33(67.3%)	0.086	Reference	0.048
	Yes	11	7(63.6%)	4(36.4%)		5.54(1.01,42.02)	
Conditioning	MAC	26	11(42.3%)	15(57.7%)	0.60	Reference	0.80
	NMA/RIC	34	12(35.3%)	22(64.7%)		0.74(0.21,2.60)	
Donor	Matched	45	17(37.8%)	28(62.2%)	1.00	Reference	1.00
	Alternative	15	6(40.0%)	9(60.0%)		0.98(0.21,4.26)	

Graft source	PBSC/UCB	48	19(39.6%)	29(60.4%)	0.75	Reference	0.88
	BM	12	4(33.3%)	8(66.7%)		0.68(0.11,3.50)	
Prior Jakafi use	No	40	18(45.0%)	22(55.0%)	0.17	Reference	0.083
	Yes	20	5(25.0%)	15(75.0%)		0.26(0.04,1.14)	

BM: bone marrow; DRI: disease risk index; HCT: hematopoietic stem cell transplant; HCT-CI: hematopoietic stem cell transplant comorbidity index; MAC: myeloablative conditioning; NMA: non-myeloablative conditioning; PBSC: peripheral blood stem cells; RIC: reduced intensity conditioning; UCB: umbilical cord blood

* Based on exact logistic regression model adjusting for female to male HCT and prior Jakafi use.

Supplemental Table S2: Multivariate Analysis

		NRM						*Overall Survival*				
		N	*6 Mo (95%CI)*	*HR (95%CI)*	*Gray test p value*	*Adjusted HR (95%CI)**	*P value**	*1 Yr (95%CI)*	*HR (95%CI)*	*Logrank p*	*Adjusted HR (95%CI)†*	*P value†*
Age at HCT, yrs	≤59	38	0.459(0.292,0.612)	Reference	0.055	Reference	0.34	0.444(0.283,0.593)	Reference	0.011	Reference	0.19
	≥60	22	0.636(0.391,0.804)	1.93(0.96,3.89)		1.46(0.67,3.19)		0.227(0.083,0.414)	2.10(1.12,3.94)		1.72(0.76,3.87)	
Recipient sex	M	39	0.553(0.379,0.696)	Reference	0.68	Reference	0.70	0.383(0.233,0.531)	Reference	0.66	Reference	0.27
	F	21	0.476(0.248,0.674)	0.85(0.43,1.68)		1.17(0.54,2.54)		0.333(0.149,0.531)	1.15(0.61,2.18)		1.46(0.74,2.86)	
Donor age, yrs	≤29	31	0.400(0.224,0.570)	Reference	0.065	Reference	0.081	0.414(0.240,0.580)	Reference	0.43	Reference	0.47
	≥30	29	0.655(0.446,0.801)	1.91(0.97,3.75)		1.89(0.93,3.86)		0.310(0.156,0.479)	1.27(0.69,2.35)		1.27(0.67,2.41)	
HCTCI	0	11	0.300(0.060,0.597)	Reference	0.13	Reference	0.12	0.455(0.167,0.707)	Reference	0.33	Reference	0.12
	1-2	21	0.667(0.409,0.832)	3.34(0.96,11.59)		3.47(0.99,12.14)		0.190(0.059,0.377)	1.76(0.70,4.45)		1.65(0.65,4.19)	
	≥3	28	0.500(0.301,0.670)	2.72(0.78,9.47)		2.40(0.69,8.29)		0.464(0.276,0.633)	1.18(0.46,2.99)		0.79(0.30,2.08)	
KPS	90-100	41	0.475(0.312,0.621)	Reference	0.13	Reference	0.12	0.389(0.242,0.533)	Reference	0.13	Reference	0.056
	≤80	19	0.632(0.364,0.811)	1.70(0.89,3.26)		1.72(0.87,3.41)		0.316(0.129,0.522)	1.58(0.83,2.99)		1.90(0.98,3.68)	
DRI	Low, Intermediate, non-Meligant	42	0.488(0.326,0.631)	Reference	0.17	Reference	0.39	0.452(0.299,0.594)	Reference	0.022	Reference	0.042
	High, Very High	18	0.611(0.337,0.800)	1.54(0.80,2.97)		1.34(0.69,2.58)		0.148(0.029,0.357)	2.02(1.06,3.84)		1.95(1.02,3.70)	

F to M HCT	No	49	0.521(0.369,0.652)	Reference	0.82	Reference	0.90	0.367(0.236,0.500)	Reference	0.80	Reference	0.84
	Yes	11	0.545(0.204,0.793)	0.89(0.36,2.21)		1.06(0.38,2.95)		0.341(0.091,0.616)	0.90(0.40,2.03)		0.92(0.40,2.11)	
Conditioning	MAC	26	0.423(0.230,0.605)	Reference	0.045	Reference	0.008	0.494(0.291,0.668)	Reference	0.026	Reference	0.046
	NMA/RIC	34	0.606(0.414,0.752)	2.01(1.06,3.82)		2.35(1.24,4.43)		0.265(0.132,0.418)	1.99(1.05,3.76)		1.92(1.01,3.64)	
Donor	Matched	45	0.467(0.315,0.605)	Reference	0.11	Reference	0.013	0.422(0.278,0.560)	Reference	0.096	Reference	0.067
	Alternative	15	0.714(0.371,0.892)	1.78(0.89,3.53)		2.22(1.18,4.16)		0.200(0.049,0.424)	1.72(0.89,3.34)		1.91(0.96,3.83)	
Graft source	PBSC/UCB	48	0.532(0.378,0.664)	Reference	0.69	Reference	0.55	0.353(0.222,0.487)	Reference	0.35	Reference	0.53
	BM	12	0.500(0.192,0.748)	0.84(0.39,1.80)		1.30(0.56,3.03)		0.417(0.152,0.665)	0.68(0.30,1.54)		0.77(0.34,1.75)	
Prior Jakafi use	No	40	0.487(0.321,0.634)	Reference	0.32	Reference	0.085	0.400(0.250,0.546)	Reference	0.45	Reference	0.89
	Yes	20	0.600(0.345,0.783)	1.39(0.73,2.63)		1.84(0.92,3.67)		0.300(0.123,0.501)	1.27(0.67,2.40)		1.05(0.52,2.13)	

BM: bone marrow; DRI: disease risk index; HCT: hematopoietic stem cell transplant; HCT-CI: hematopoietic stem cell transplant comorbidity index; MAC: myeloablative conditioning; NMA: non-myeloablative conditioning; PBSC: peripheral blood stem cells; RIC: reduced intensity conditioning; UCB: umbilical cord blood

* Based on multivariable Fine and Gray model adjusting for conditioning intensity and donor type.
† Based on multivariable Cox regression model adjusting for DRI and conditioning intensity.
